# Notes on the blood-feeding behavior of *Aedes albopictus *(Diptera: Culicidae) in Cameroon

**DOI:** 10.1186/1756-3305-5-57

**Published:** 2012-03-21

**Authors:** Basile Kamgang, Elysée Nchoutpouen, Frédéric Simard, Christophe Paupy

**Affiliations:** 1 Institut de Recherche pour le Développement (IRD), UMR MIVEGEC (UM1-CNRS 5290-IRD 224), team BEES, P.O. Box 64501, Montpellier 34394, France; 2 Laboratoire de Recherche sur le Paludisme, Organisation de Coordination pour la lutte contre les Endémies en Afrique Centrale (OCEAC), P.O. Box 288, Yaoundé, Cameroon; 3 Institut Pasteur de Bangui, P.O. Box 923, Bangui, Republic of Central Africa; 4 Centre International de Recherches Médicales de Franceville (CIRMF), P.O. Box 769, Franceville, Gabon

**Keywords:** *Aedes albopictus*, Feeding behavior, Host preference, Nychthemeral activity, Cameroon

## Abstract

**Background:**

The invasive mosquito *Aedes albopictus *is often considered a poor vector of human pathogens, owing to its catholic feeding behavior. However, it was recently incriminated as a major vector in several Chikungunya epidemics, outside of its native range. Here we assessed two key elements of feeding behavior by *Ae. albopictus *females in Yaoundé, Cameroon, Central Africa. Host preference was explored and the human-biting activity of females was monitored over 24 h to determine periods of maximum bite exposure.

**Findings:**

Analysis of ingested blood in outdoor-resting females showed that *Ae. albopictus *preferentially fed on humans rather than on available domestic animals (95% of the blood meals contained human blood). Our results further showed that *Ae. albopictus *is a day-biting species in Yaoundé, with a main peak of activity in the late afternoon.

**Conclusion:**

This is the first report on the feeding behavior of *Ae. albopictus *in Central Africa. The species is highly aggressive to humans and might therefore be involved in human-human virus transmission in this setting.

## Background

*Aedes albopictus *(Skuse, 1894) is an invasive mosquito species that originated in Asian forests [1] and expanded to temperate and tropical regions of America, Africa and Europe, mainly during the last three decades [2]. *Aedes albopictus *is widely considered as a secondary vector of human arboviruses such as dengue virus (DENV), because it is thought to preferentially feed on animals rather than humans, contrary to the highly anthropophilic *Aedes aegypti *(L., 1762) [3]. Yet, *Ae. albopictus *has been incriminated as a primary vector in recent chikungunya virus (CHIKV) epidemics in the Indian Ocean, Central Africa and Europe [4-6], suggesting sustained man-vector contact in these settings. In Cameroon (Central Africa), *Ae. albopictus *was first recorded in the early 2000s [7] and rapidly spread throughout the south of the country [8]. In Cameroonian urban centers such as Yaoundé, the species now pullulates [9] and is gradually replacing autochthonous *Ae. aegypti *populations. Concomitant with the spread of *Ae. albopictus *in Central Africa, an increase in DENV and CHIKV outbreaks was reported in a number of countries, including Cameroon [10], Gabon [5,11] and the Republic of Congo [12]. Indeed, Cameroonian *Ae. albopictus *populations were shown to be orally susceptible to DEN-2 virus and CHIKV infection [5], and the species was recognized as the main vector of both viruses in 2007 in Libreville, Gabon [5,11].

Although *Ae. albopictus *preferentially bites mammals [3], females can also feed on most groups of vertebrates, both cold- and warm-blooded, including reptiles, birds and amphibians [13]. Such feeding plasticity, which has been found to vary according to the geographic origin of mosquito populations [14], maximizes the fitness (fecundity and survival) of *Ae. albopictus *and enhances the risk that it may propagate zoonotic pathogens from wildlife or domestic animals to humans [15]. In addition to host preferences, the intensity of human-vector contact and, therefore, the risk of pathogen transmission, is modulated by the biting period(s) along the nychthemere, which impacts on host availability. Although geographic variations have been recorded in the biting rhythm of *Ae. Albopictus *females, the species usually bites outdoors and during daytime, with two main peaks of activity in the morning and early evening [3,4,14]. To our knowledge, there are no published data on the feeding behavior of *Ae. albopictus *in Central Africa. In order to fill this gap, we conducted a study of *Ae. albopictus *host preferences and biting activity in Yaoundé, Cameroon.

## Methods

Mosquitoes were collected from two sites in the periphery of Yaoundé, the capital city of Cameroon: "Club France" (N3°50'28", E11°30'2") in the Efoulan district and "Club hippique" (N3°54'05", E11°29'53") in the Ntougou district. "Club France", a leisure center (tennis, swimming pool, banquets, etc.;), is surrounded by dense vegetation which provides potential resting and oviposition sites for *Aedes *mosquitoes. "Club hippique", an equestrian center, is also surrounded by dense vegetation. The horses (N > 25) provide potential blood sources for mosquitoes. Additionally, both sites are surrounded by human dwellings with domestic animals such as dogs, pigs and chickens.

Field collections were conducted from April to early July 2009, during the rainy season when mosquito densities are highest. To determine the feeding preferences of *Ae. albopictus*, wild blood-fed females were caught in both study sites. The low vegetation that constitutes resting sites was stirred up to disturb mosquitoes, and flying females were caught by sweep-netting. Each capture was done by a single collector and lasted 30 min. Collected mosquitoes were individually transferred to 10-mL glass vials using a mouth aspirator; they were identified morphologically by reference to standard keys [16] and stored at -20°C until blood meal analysis. The abdomen of freshly-fed *Ae. albopictus *and *Ae. aegypti *females was dissected out and used as a template for blood-meal analysis by ELISA [17], using anti-human, -horse, -bird, -reptile, -swine, -sheep and -dog antibodies (Sigma Aldrich, St. Louis, MO). To explore the biting activity of *Ae. albopictus*, blood-seeking females were trapped outdoors over entire 24-hour periods using a double-net device baited with a human volunteer, as described in Delatte et al. [14]. The experiment was repeated eight times in "Club France" only, between April and July 2009.

## Results and discussion

In "Club France", a total of 537 resting *Aedes *females were harvested during 46 capture sessions, of which 479 were *Ae. albopictus *(including 139 freshly fed) and 58 *Ae. aegypti *(including 6 freshly fed). Twenty-one capture sessions in "Club hippique" recovered 77 *Ae. albopictus *females (including 31 freshly fed) and no *Ae. aegypti*. Males of either species were observed but not collected, as well as other mosquito genera such as *Culex*. ELISA analyses indicated that *Ae. albopictus *specimens had primarily fed on human blood, as about 95% of dissected stomachs contained human blood in the two sites (Table 1). Mixed meals of human and reptile or pig blood were also observed. All 6 *Ae. aegypti *females collected from "Club France" had fed on human blood. These results conflict with the assumption that *Ae. albopictus *is mainly zoophilic [2,3,13], and are consistent with observations made in Thailand [18], North Carolina (United States) [19], Italy [20] and La Réunion [14]. Our results are all the more convincing as animals were available in both study sites, showing that *Ae. albopictus *prefers to feed on humans, at least in Yaoundé. This propensity of *Ae. albopictus *females to feed on humans in urban areas of Cameroon is of concern, as it suggests a risk of human-human pathogen transmission. Moreover, the observation of blood meals from pigs and reptiles, and especially mixed animal-human meals, although few in number, confirms that this species could act as a bridge vector for zoonotic pathogens. Furthermore, in about 5% of cases the host species could not be identified, suggesting either advanced digestion of the blood or the existence of unidentified animal hosts such as rodents.

**Table 1:** Ingested-blood analysis in *Ae.albopictus *females from sites in Yaoundé, Cameroon

Host	Number of specimens analyzed (% positive)	
	***Club France***	***Club hippique***

Human	130 (93.5)	26 (83.9)

Reptile	1 (0.7)	0

Horse	0	0

Chicken	0	0

Dog	0	0

Sheep	0	0

Swine	0	0

Human-Reptile	2 (1.5)	0

Human-Swine	0	3 (9.7)

No-call^a^	6 (4.3)	2 (6.4)

**Total**	139 (100)	31 (100)

^a ^No reaction with antibodies to any of the tested hosts.

**Figure 1:**
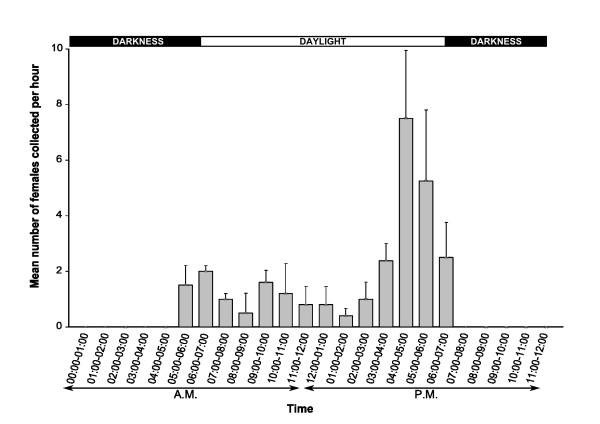
**Daily dynamics of host-seeking activity in *Ae. albopictus *females from Yaoundé, Cameroon (April-July 2009)**. Vertical bars represent standard deviation.

Mosquito collection using a double-net device demonstrated that *Ae. albopictus *females feed during daytime from 5:00 a.m. to 7:00 p.m. with a main peak from 3:00 p.m. to 7:00 p.m (Figure 1). These results are consistent with the known biology of *Ae. albopictus *outside Africa [3,14,21]. Although *Ae. albopictus *is sometimes observed indoors, it is generally considered as exophilic and exophagic [4]. In their recent work in La Réunion Island (France, Indian Ocean), using a similar double-net collection device placed indoors and outdoors, Delatte et al. [14] estimated that 89% of mosquitoes were exophagic. Our results, based solely on outdoor collections, therefore, likely reflect the major biting pattern of *Ae. albopictus *females in this area. However, further studies are required to fully explore the feeding behavior of invasive populations of *Ae. albopictus *and to assess their role in the emergence and spread of arbovirus epidemics in Africa and elsewhere, including areas where *Ae. aegypti *is the dominant species.

## Conclusion

This study, which provides the first data on the feeding behavior of *Ae. albopictus *in Central Africa, demonstrates that this species readily bites humans in urban settings and might therefore play a major role in human-human transmission of emerging arboviruses such as DENV and CHIKV. Detailed knowledge of the feeding behavior of this vector species, such as the rhythm of females' biting activity, is needed to develop and implement efficient personal protection tools against *Ae. albopictus*, especially in newly colonized areas.

## Competing interests

The authors declare that they have no competing interests.

## Authors' contributions

BK and CP designed the study and monitored its implementation. BK and EN conducted the field work. BK analyzed the data. BK and CP wrote the manuscript which was critically revised by FS. All authors read and approved the final manuscript.
